# Maternal identity measurement based on the experiences of mothers with infants: a methodological study

**DOI:** 10.4069/whn.2025.03.08

**Published:** 2025-03-28

**Authors:** Sun jung Park, Eun young Choi

**Affiliations:** 1 Department of Nursing, Sahmyook Health University, Seoul, Korea; 2 Department of Nursing, Kyungdong University, Seoul, Korea

**Keywords:** Infants, Maternal identity, Measurement tool, Mothers

## Abstract

**Purpose:**

This study aimed to develop and validate a tool to measure maternal identity in mothers caring for infants.

**Methods:**

This methodological study involved two stages: tool development and evaluation. A preliminary 35‐item tool was created and refined to 22 items and administered to 300 mothers with infants. Data were collected between May and June 2024. Descriptive statistics, Pearson correlation, exploratory factor analysis (using Varimax rotation), and confirmatory factor analysis (via AMOS 21.0) were performed. Convergent validity was evaluated using an adapted version of the Maternal Confidence Questionnaire. Internal consistency (Cronbach’s α) and split‐half reliability (Guttman split) were also assessed.

**Results:**

Exploratory factor analysis identified four sub-factors consisting of 17 items: “warmth type” (seven items), “best effort type” (five items), “indifference type” (three items), and “preparation type” (two items). The content validity index was .86, and construct validity accounted for 59.5% of the variance. Factor loadings ranged from 0.57 to 0.80. Convergent validity was confirmed (r=.70, *p*<.001). Internal consistency was high, with Cronbach’s α values between .85 and .90 and split-half reliability coefficients between .82 and .91. The final tool, named the Maternal Identity Measurement for Mothers with Infants, comprises 17 items rated on a 5-point scale (1–5), with higher scores (possible range, 17–85) indicating a stronger maternal identity.

**Conclusion:**

The developed tool demonstrated strong reliability and validity for assessing maternal identity in mothers of infants. It offers a structured approach for evaluating maternal identity in the context of infant care and has practical implications for maternal health research and interventions.

## Introduction

In modern Korean society, there is an increasing recognition of the significant role of mothers, particularly amid low birth rates. Maternal identity extends beyond child-rearing; it plays a vital role in shaping a woman’s self-awareness, psychological well-being, and the quality of her interactions with her children [1]. According to Statistics Korea’s population trends report from June 2023, the number of births in the second quarter of 2023 was 50,087—a 6.8% decrease (4,062 fewer births) compared to the same period the previous year. This data underscores the persistent low birth rate issue [2]. Additionally, the Organisation for Economic Co-operation and Development (OECD) reports that South Korea remains the only member country with a total fertility rate below 1, recording 0.78 in 2022—well below the OECD average of 1.59. This stark disparity highlights the severe decline in South Korea’s birth rate [2,3]. The low birth rate phenomenon not only reduces fertility rates but also poses long-term societal challenges, such as demographic shifts, diminished economic growth potential, and increased strain on social care systems. Consequently, South Korean society must implement strategies to enhance the sense of happiness associated with childbirth and parenting, while empowering women to develop a stronger, more positive maternal identity.

Maternal identity encompasses more than just parenting skills; it includes a mother’s self-awareness, psychological stability, and positive self-image. It is essential for a mother’s personal growth and well-being and serves as a foundation for promoting healthy development in young children. Through positive and nurturing interactions, a well-developed maternal identity creates a supportive environment that significantly contributes to a child’s emotional and psychological growth [4]. Maternal identity involves reconstructing one’s self and developing self-awareness while fulfilling the maternal role. This process is crucial not only for postpartum adaptation but is also enhanced through ongoing interactions with the child [5]. Infancy represents a critical period for overall development, during which a mother’s role greatly influences her child’s growth and development [6]. Recent rapid social, economic, and cultural changes in Korea—especially the rise in dual-income families and increased workforce participation by women—have transformed traditional maternal roles [7,8]. Although many mothers adapt successfully, some experience overwhelming stress and burdens that lead to negative emotions such as helplessness, guilt, conflicting feelings, depression, and anger [9-11]. These emotions may impair the sensitivity and warmth of interactions between mothers and their children [12]. Although various tools to measure maternal identity have been developed and are widely used both domestically and internationally, many rely on specific theoretical frameworks [11,12] and fail to adequately consider the evolving social [13], cultural [14,15], and regional contexts [16,17].

In South Korea, rapid societal changes, such as the increase in dual-income households and greater female workforce participation, have profoundly reshaped the conceptualization and practice of maternal roles. Despite these shifts, there remains a significant gap in measurement instruments that address these evolving contextual dynamics. Existing tools primarily focus on mother-fetus interactions during pregnancy or early postpartum adaptation, limiting their ability to capture the multifaceted nature of maternal identity as experienced by modern mothers. Moreover, these instruments do not sufficiently account for the unique social and cultural contexts or the emotional experiences of Korean mothers. Therefore, a new approach is needed to better understand and measure the maternal experiences of contemporary Korean women.

The purpose of this study is to develop a tool specifically designed to assess the maternal identity of mothers caring for their infants.

## Methods

Ethics statement: This study was approved by the Institutional Review Board (IRB) of Kyungdong University (IRB-1041455-202404-HR-008-01). Informed consent was obtained from the participants.

### Research design

**Figure 1.**
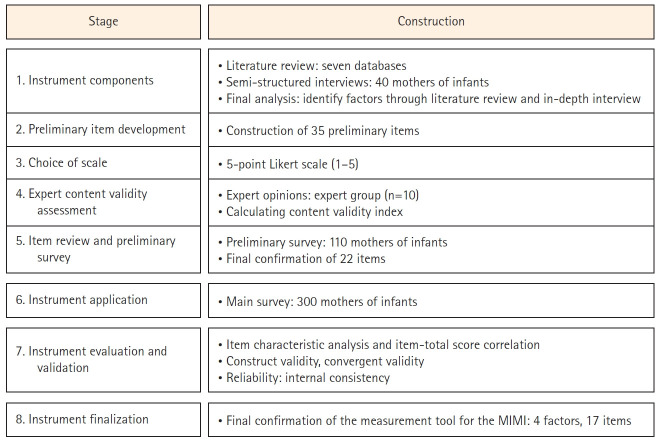
Development process of the Maternal Identity Measurement of Mothers with with Infants (MIMI).

This methodological study was designed to develop a tool for assessing maternal identity in mothers caring for infants and to test its validity and reliability. This study followed the eight-step tool development process outlined by DeVellis [18]. The specific research process is as follows (Figure 1).

#### Step 1: instrument components

The concept of maternal identity was precisely defined, and the target population was identified as mothers with infants. This determination was based on a comprehensive review of relevant literature from both domestic and international sources. The literature review was conducted using databases such as KISS (Korean Studies Information Service System), RISS (Research Information Sharing Service), KCI (Korea Citation Index), Clinical Key for Nursing, PubMed, EMBASE, and ProQuest to locate relevant published articles and dissertations. Key search terms included “maternal identity,” “maternal identity in infancy,” and related phrases. Initial findings guided the review. Existing scales on maternal identity, as documented by Kim and Hong [17], Mercer and Walker [11], Song and Ahn [13], and Song et al. [14], were used to formulate a comprehensive set of questions. Additionally, semi-structured interviews with 40 mothers of infants were conducted to further enrich and contextualize the questionnaire pool.

#### Step 2: preliminary item development

Preliminary questions were formulated based on the aspects of maternal identity identified through the experiences of mothers with infants, as determined by the literature review and in-depth individual interviews.

#### Step 3: choice of scale

A 5-point Likert scale was employed in this study to eliminate neutral responses. This approach provided clearer insight into respondents’ attitudes and reduced potential distortion in interpreting the results.

#### Step 4: expert content validity assessment

To ensure that the preliminary instrument effectively measured the intended content, content validation was performed with an expert panel. Following Lynn’s recommendations for content validation studies [18], the panel consisted of 10 experts: two professors specializing in maternal nursing, one professor in pediatric nursing, two maternity ward nurses, one obstetrician, three mothers with young children, and one professor of statistics. The item-level content validity index (I-CVI) was calculated as the proportion of experts rating each item as relevant. Items with an I-CVI of .80 or higher were retained [19]. Additionally, the scale-level content validity index (S-CVI) was computed by averaging the I-CVI scores across all items and was considered acceptable if it exceeded .90. Preliminary items meeting these criteria were retained, and insights from the expert consultation were incorporated to refine and expand the questionnaire.

#### Step 5: item review and main survey

A preliminary survey was conducted from January 10 to January 20, 2024, involving 120 mothers of infants from three cities—Seoul, Gangwon Province, and Gyeonggi Province—to evaluate the questionnaire items. Participants provided feedback on the initial questions and demographic characteristics. Based on this feedback, the survey was revised to improve question clarity, response time, questionnaire structure, and item length, with additional content incorporated as needed. Subsequently, a Korean literature expert with over 5 years of university teaching experience evaluated the overall grammar and vocabulary of the survey items. The preliminary instrument’s reliability was assessed using Cronbach’s α and split-half reliability. Construct validity was evaluated through item analysis and factor analysis, which included calculating item-total correlation coefficients and Cronbach’s α if an item was removed. Five of the initial 27 items were eliminated based on these analyses.

#### Step 6: instrument application

Participants were selected based on the following inclusion criteria: mothers of infants who were free from general illnesses or physical deformities, were not part of multicultural families, could communicate effectively, and provided informed consent. The study’s purpose and procedures were explained both verbally and in writing, and voluntary consent was obtained prior to participation. Based on previous research on sample sizes suitable for factor analysis [20], the target sample size was set at 300, accounting for an estimated 10% dropout rate. Ultimately, 311 participants were recruited using convenience sampling. After excluding 11 invalid responses, the final dataset comprised 300 valid responses. The survey was conducted from May 1 to June 20, 2024. Data collection took place after obtaining permission from institutions such as local health centers, cultural centers, and pediatric hospitals through official correspondence. As in the preliminary survey, mothers visiting these institutions—such as health centers, daycare centers, and pediatric hospitals—were approached for participation. Institutional directors facilitated survey cooperation, and the primary investigator, along with two research assistants, conducted the surveys in person. To encourage participation, respondents were provided with small token gifts.

#### Step 7: instrument evaluation

Descriptive statistics were used for item analysis, and exploratory factor analysis (EFA) was conducted to assess construct validity. Convergent validity was evaluated using an adapted version of the Maternal Confidence Questionnaire, originally developed by Zahr [21]. Internal consistency, as a measure of the reliability of the instrument’s items, was determined by calculating Cronbach’s α.

#### Step 8: instrument finalization

The instrument was refined by removing items that compromised its validity and reliability. After these adjustments, the scale was finalized and designated as the Maternal Identity Measurement for Mothers with Infants (MIMI).

### Data analysis

The reliability and validity of the developed tool were evaluated using IBM SPSS for Windows ver. 21.0 (IBM Corp., Armonk, NY, USA) and AMOS ver. 21.0 (IBM Corp., Armonk, NY, USA). Demographic characteristics were analyzed using descriptive statistics, including percentages, frequencies, means, and standard deviations. Pearson correlation analysis was employed for item analysis where applicable. EFA was conducted using the Varimax rotation method, while confirmatory factor analysis was performed using AMOS. Internal consistency, measured by Cronbach’s α, and split-half reliability, assessed using the Guttman split-half method, were calculated for the overall scale and for each factor to ensure reliability.

## Results

### Step 1: instrument components

Four dimensions—the warmth type, best effort type, indifference type, and preparation type—were identified through a literature review and in-depth interviews. These dimensions informed the development of a maternal identity measurement tool based on the experiences of Korean mothers with infants.

### Step 2: preliminary items

Based on the identified dimensions, attributes, and indicators, a total of 22 preliminary items were developed across the four dimensions. Specifically, nine items pertained to the warmth type, 6 items to the best effort type, 4 items to the indifference type, and three items to the preparation type.

### Step 3: scale selection

The study employed a 5-point Likert scale, a common method for measuring maternal identity, to assess the experiences of Korean mothers with infants. The scale ranged from 1 (“not at all”) to 5 (“very much”), intentionally designed to avoid neutral responses.

### Step 4: expert content validity

The I-CVI values ranged from .90 to 1.00. The S-CVI, calculated by averaging the I-CVI values, yielded a score of .95. This exceeds the established threshold of .90, confirming the instrument’s content validity.

### Step 5: item review and preliminary survey

Before the main study, a preliminary survey was conducted with 110 mothers of infants from three cities—Seoul, Gangwon Province, and Gyeonggi Province—to evaluate the questionnaire items. Completion time ranged from 10 to 20 minutes, with an average of 15 minutes. The survey items received an overall comprehensibility rating of 3.90±0.84, a questionnaire structure rating of 4.14±0.64, and appropriateness of item length rating of 3.67±0.44 (Table 1). Following a review by a Korean language expert, minor revisions were made to improve grammar and vocabulary. For example, “I am mature as a mother to my children” was revised to “As a mother, I show mature behavior to my child” to enhance clarity. This process finalized a set of 22 items. The reliability of the preliminary instrument was tested using Cronbach’s α coefficient and split-half reliability, while construct validity was assessed through item analysis and factor analysis. The reliability of the preliminary instrument was shown by a Cronbach’s α value of .92, while the Cronbach’s α value for split-half reliability was .90. The assessment of construct validity through item analysis and factor analysis led to the removal of five items from the initial 27.

### Step 6: main survey findings

#### General characteristics of the participants

A total of 300 subjects participated in the survey. There was a slight predominance of female infants (168 cases, 56.0%), and the majority of children were reported to be in good health (286 cases, 95.3%). All caregivers had attained at least a high school education, and a significant proportion (229 cases, 76.3%) were not employed. Among employed caregivers, occupations included professional, office, service, and managerial roles. The duration of marriage was at least 5 years, and over 90% of participants reported an average monthly income of 2 million Korean won (approximately 1,700 US dollars), which is comparable to the average monthly household income in Korea for 2024 [2].

### Step 7: instrument evaluation and validation

#### Analysis of item characteristics and item-total correlation

The mean values for the items ranged from 3.00 to 4.52, and standard deviations ranged from 1.15 to 1.67, which were within acceptable limits. Additionally, skewness and kurtosis for all items were within ±2.00, indicating conformity with the assumption of normality. The 22 items demonstrated satisfactory performance, with item-total correlation coefficients ranging from .30 to .80 and an overall Cronbach’s α of .92, supporting the retention of all items for further analysis [22].

#### Construct validity: exploratory factor analysis

EFA was conducted on the 22 selected items in four iterations to identify factor structures and their relationships with associated items. The Kaiser-Meyer-Olkin measure of sampling adequacy was .86, exceeding the recommended threshold of .80 [23]. Bartlett’s test of sphericity was statistically significant (*χ*^2^=2,532.56, df=231, *p*<.001), confirming the data’s suitability for factor analysis. Four factors with eigenvalues greater than 1.0 were extracted, accounting for 59.5% of the total variance. Factor loadings ranged from .57 to .80, and communalities ranged from .43 to .87, all exceeding the predetermined thresholds (Table 2). As a result, 17 items were retained and grouped into four factors: “warmth type” (seven items), “best effort type” (five items), “indifference type” (three items), and “preparation type” (two items).

#### Convergent validity test results

Convergent and discriminant validity were assessed by examining the correlations between the 17 items of the developed scale and related variables. The results revealed a strong positive correlation between the scores of the developed tool and the Maternal Role Confidence scores (r=.70, *p*<.001), as well as a low correlation with Parenting Stress Index scores (r=.33, *p*<.001). These findings confirm the convergent and discriminant validity of the developed scale.

#### Reliability test: internal consistency evaluation

**Figure 2.**
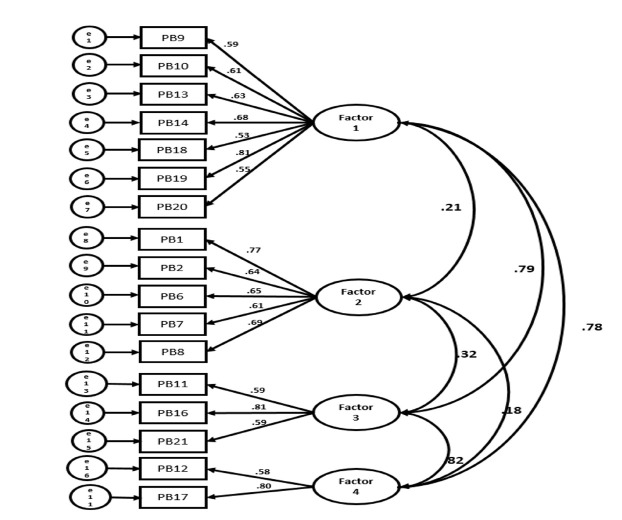
Confirmatory factor analysis.

The internal consistency of the final 17-item scale and its individual factors was assessed using Cronbach’s α and Guttman split-half reliability. The results are summarized in Table 3 and Figure 2.

For Factor 1 (seven items), Cronbach’s α was .90 and the Guttman split-half reliability coefficient was .91. For Factor 2 (five items), Cronbach’s α was .89 and the Guttman split-half reliability coefficient was .87. For Factor 3 (three items), Cronbach’s α was .85 and the Guttman split-half reliability coefficient was .82. For Factor 4 (two items), Cronbach’s α was .85, and the Guttman split-half reliability coefficient was. 86.

### Step 8: instrument finalization

The tool was finalized with 17 items, categorized into four factors: warmth type (seven items), best effort type (five items), indifference type (three items), and preparation type (two items). The final tool employs a 5-point Likert scale, ranging from 1 (“not at all”) to 5 (“very much”). Total scores range from 17 to 82, with higher scores indicating a stronger sense of maternal identity among mothers of infants (Supplementary Material 1).

## Discussion

This study developed a maternal identity measurement tool specifically for mothers caring for infants. The innovative and validated tool lays the groundwork for tailored interventions to enhance maternal identity in caregiving contexts. The tool comprises several components. The first factor, “warmth type,” primarily captures expressions of warm love and recognition. This finding aligns with previous research [24], which linked maternal identity in child-rearing to elements such as “love,” “calmness,” and “warmth.” Similarly, another study [25] identified affection as a predominant factor in maternal parenting behavior. These findings suggest that affectionate parenting behaviors are increasingly prevalent among Korean mothers of infants and that the “warmth type” factor effectively reflects maternal identity in Korea.

Compared to previously developed tools that assess maternal identity in mothers of children of various age groups, the tool developed in this study is specifically tailored to capture the unique characteristics and experiences of mothers with infants. For example, the “warmth type” factor emphasizes affectionate expressions and recognition, which are especially significant in infant care. This focus differentiates it from tools designed for mothers of older children and allows for a more accurate assessment of maternal identity specific to this demographic.

The second factor, termed “best effort type,” reflects a proactive response to the child’s needs, characterized by keen interest and prompt engagement in the child’s activities. This is consistent with findings from a previous study [24] that identified behaviors such as “taking care of my child before attending to my own needs after childbirth” and “reacting immediately when the baby cries despite sleep deprivation.” In addition, another study [26] highlighted the experiences of working parents as “novice caregivers who do their best in their parenting roles,” which closely aligns with the findings of this study. These results suggest that even inexperienced mothers exert significant effort to fulfill their maternal roles, demonstrating their commitment to parenting.

The third factor represents a lack of effort in creating a nurturing environment for children, characterized by insufficient time, resources, interest, or investment in the child’s well-being. These findings resonate with a study [27] that demonstrated how a mother’s physical and psychological health significantly influences her relationship with her infant.

The fourth factor, “preparation type,” pertains to the readiness to provide children with optimal mental, physical, and financial support by consistently ensuring that the child’s needs are met. This aligns with findings from a study [28], which revealed that higher levels of maternal preparedness for child-rearing and self-differentiation are associated with reduced parental frustration and anxiety.

This study explores maternal identity among mothers raising infants in Korea and emphasizes its significance by developing and validating a culturally sensitive measurement tool. The instrument was developed within the sociocultural context of Korean mothers, and its applicability to other cultural contexts remains unknown. Additionally, the measurement tool is specifically designed for the infant stage and has not been adapted for other parenting phases. One of the study’s strengths is its provision of valuable insights through a culturally relevant framework that addresses factors such as delayed maternal age and the challenges of dual-career households. The findings highlighted an increase in the average maternal age to 41 years, primarily due to delayed first marriages in Korea. The study underscores the importance of examining how maternal identity is influenced by demographic factors (e.g., age, education, income, and marital duration) as well as child-related factors (e.g., age and health). Given societal trends such as delayed marriage and declining fertility rates, fostering maternal identity is essential to help mothers adapt to evolving socio-economic and cultural dynamics.

Additionally, the study provides a foundational understanding of maternal identity and highlights the importance of extending the tool’s application to different parenting stages and cultural contexts.

## Figures and Tables

**Table 1.** Item analysis results (N=110)

Category	Range (minute)	Mean±SD
Time required to complete the survey	4–25	15±4.10
Level of clarity of the questions	1-5	3.90±0.84
Appropriateness of layout	1-5	4.14±0.64
Appropriateness of question length	1-5	3.67±0.44

**Table 2.** Factor analysis results (N=300)

Item No.	Item contents	Factor loading			
		Factor 1	Factor 2	Factor 3	Factor 4
4	I’m happy when I am with my child.	0.65	0.09	–0.06	0.13
5	My child is the prettiest regardless of how he or she looks.	0.70	0.24	–0.01	0.01
9	I feel good when I see my child.	0.81	0.01	0.02	0.11
10	I have a special feeling for my child.	0.85	0.00	0.09	0.11
13	I cherish my child.	0.67	0.03	0.06	-0.03
14	I love my child.	0.87	0.04	–0.02	0.15
18	I don’t know how time flies when I’m with my kids.	0.44	–0.10	–0.04	0.21
19	I’m saddest when my child is sick.	0.81	–0.09	0.16	0.02
20	I most like to spend time with my child.	0.85	0.00	0.04	-0.05
1	As a mother, I react quickly to everything about my children.	0.02	0.76	0.14	-0.02
2	As a mother, I’m kind to my child.	–0.06	0.67	–0.06	-0.10
6	As a mother, I do my best when dealing with my child.	0.10	0.68	–0.03	-0.08
7	As a mother, I show mature behavior to my child.	0.10	0.64	–0.00	0.14
8	As a mother, I’m careful about everything I do for my child.	0.02	0.79	0.12	-0.01
15	I do my best for my child’s health.	0.14	0.88	–0.10	-0.00
11	I have no feelings for my child.	0.17	–0.01	0.74	0.14
16	I’m not interested in my child.	0.17	0.07	0.67	0.02
21	I take care of my child with a sense of duty.	0.07	–0.04	0.71	0.06
22	I have a lot of ups and downs when dealing with my child.	0.07	–0.01	0.68	0.05
3	I’m physically healthy as a mother.	0.10	0.01	–0.05	0.69
12	As a mother, I have a wealth of knowledge about parenting.	0.01	–0.01	0.01	0.62
17	I’m mentally healthy as a mother.	0.10	0.08	–0.02	0.66
Eigenvalue		7.14	3.48	1.30	1.16
Explained variance (%)		32.46	15.85	5.91	5.29
Accumulative variance (%)		32.46	48.32	54.24	59.53
Kaiser-Meyer-Olkin			0.87		
Bartlett’s test of sphericity		Chi-square statistic=2,532.56, degrees of freedom=231, *p*<.001			

**Table 3.** Confirmatory factor analysis (N=300)

Factor	Standardized χ²	SE	CR	*p*-value
Factor 1 (warmth type)	0.59	-	-	<.001
	0.61	0.24	4.22	<.001
	0.63	0.14	4.32	<.001
	0.68	0.15	4.58	<.001
	0.53	0.33	3.80	<.001
	0.81	0.25	5.16	<.001
	0.55	0.33	3.90	<.001
Factor 2 (best effort type)	0.77	-	-	<.001
	0.64	0.15	4.76	<.001
	0.65	0.14	4.83	<.001
	0.61	0.12	4.54	<.001
	0.69	0.13	5.10	<.001
Factor 3 (indifference type)	0.59	-	-	<.001
	0.81	0.24	5.70	<.001
	0.59	0.27	4.61	<.001
Factor 4 (preparation type)	0.58	-	-	<.001
	0.80	0.20	5.06	<.001

## Supplementary materials

Further details on supplementary materials are presented online (available at https://doi.org/10.4069/whn.2025.03.08).

## References

[1] Park S, Yu G (2024). Changes in parents’ perceptions of work-family strains, gains, and their effects on stress and life satisfaction before and after COVID-19: applying the latent growth model and actor-partner interdependence model (APIM). Korean J Child Care Educ Policy.

[2] Statistics Korea (2022). 2022 Population trends in Korea [Internet]. https://kostat.go.kr/board.es?act=view&bid=11773&list_no=427187&mid=a20108100000&utm_source.

[3] Organisation for Economic Co-operation and Development (OECD) (2023). Fertility rates in OECD countries: statistical report [Internet]. https://data.oecd.org/pop/fertility-rates.htm.

[4] Bicking Kinsey C, Hupcey JE (2013). State of the science of maternal-infant bonding: a principle-based concept analysis. Midwifery.

[5] Mercer RT (2004). Becoming a mother versus maternal role attainment. J Nurs Scholarsh.

[6] Shin SL (2017). Study on identity change experience of women in early nurture period through group narrative therapy -Focusing on motherhood ideology [dissertation].

[7] Park HJ, Moon HJ (2013). Maternal attachment to infants, depression and social support for mothers with infants and their effects on parenting efficacy. Korean J Hum Dev.

[8] Hwang HS, Lee HY, Lee GH (2008). Child development and education.

[9] KOSTAT (2018). Raising 1-3 years old children [Internet]. https://sri.kostat.go.kr/board.es?act=view&bid=0060&list_no=426078&mid=b10105000000&nPage=1&ref_bid=&tag=.

[10] Chae YS (2005). Adaptation of maternal roles and postpartum depression of primiparas during early postpartum period [master’s thesis].

[11] Mercer RT, Walker LO (2006). A review of nursing interventions to foster becoming a mother. J Obstet Gynecol Neonatal Nurs.

[12] Chae MY, Hwang MS (2011). The effects of home care nursing based maternal role strengthening programs on the maternal identity and confidence of maternal role on first-time mothers. J Korean Acad Soc Home Care Nurs.

[13] Song JE, Ahn JA (2013). Effect of intervention programs for improving maternal adaptation in Korea: systematic review. Korean J Women Health Nurs.

[14] Song JE, Chae HJ, Park BL (2015). Experiences of sanhujori facility use among the first time mothers by the focus group interview. Korean J Women Health Nurs.

[15] Osgood CE, Suji GJ, Tannenbaum PH (1957). The measurement of meaning.

[16] Walker LO (1977). Investigating the semantic properties of two concepts [master’s thesis].

[17] Kim HW, Hong KJ (1996). Development of a maternal identity scale for pregnant women. J Korean Acad Nurs.

[18] DeVellis RF (2017). Scale development: theory and applications.

[19] Lincoln YS, Guba EG (1985). Naturalistic inquiry.

[20] Comrey AL (1988). Factor-analytic methods of scale development in personality and clinical psychology. J Consult Clin Psychol.

[21] Zahr LK (1991). The relationship between maternal confidence and mother-infant behaviors in premature infants. Res Nurs Health.

[22] Hair JF, Black WC, Babin BJ, Anderson RE (2010). Multivariate data analysis: a global perspective.

[23] Kang H (2013). A guide on the use of factor analysis in the assessment of construct validity. J Korean Acad Nurs.

[24] Shin HS, Park SY, Park SJ, Go GY, Park BS (2021). Maternal identity perceived by mothers with infancy children: focused on the application of content analysis technique. J Korean Nurs Res.

[25] Park SJ, Kang KA (2015). Development of a measurement instrument for parenting behavior of primary caregivers in early childhood. J Korean Acad Nurs.

[26] Ha MS, Lee SB (2009). The influence of the children-perceived parents’ rearing attitude and self-elasticity to adjust to school life. Korean J Child Educ.

[27] Song JE, Ko JM (2016). Influencing factors on maternal role adjustment among the primipara women in the first year after childbirth. Korean J Soc Matern Child Health.

[28] Kim HS (2008). The relationship among mothers’ clinging, parenting-efficacy and preschoolers’ emotional intelligence. J Future Early Child Educ.

